# Incidence, risk factors and evolving treatment of severe retinopathy of prematurity in China: a retrospective multicenter cohort study in 70 neonatal intensive care units

**DOI:** 10.3389/fmed.2025.1652727

**Published:** 2025-09-18

**Authors:** Xuting Chen, Yu Xu, Yanchen Wang, Juan Du, Falin Xu, Xinyue Gu, Yun Cao, Jianhua Sun, Mingyan Hei, Shoo Lee, Hongping Xia, for the Chinese Neonatal Network

**Affiliations:** ^1^Department of Neonatology, Xinhua Hospital Affiliated to Shanghai Jiao Tong University School of Medicine, Shanghai, China; ^2^Department of Ophthalmology, Xinhua Hospital Affiliated to Shanghai Jiao Tong University School of Medicine, Shanghai, China; ^3^NHC Key Laboratory of Neonatal Diseases (Fudan University), Children's Hospital of Fudan University, National Children's Medical Center, Shanghai, China; ^4^Departments of Obstetrics & Gynecology, and Health Research Methods, Evidence & Impact, McMaster University, Hamilton, ON, Canada; ^5^Neonatal Center, Beijing Children's Hospital, Capital Medical University, National Children's Medical Center, Beijing, China; ^6^Department of Neonatology, The Third Affiliated Hospital of Zhengzhou University, Zhengzhou, China; ^7^Department of Neonatology, Children's Hospital of Fudan University, National Children's Medical Center, Shanghai, China; ^8^Department of Neonatology, Shanghai Children's Medical Center, Shanghai Jiao Tong University School of Medicine, Shanghai, China; ^9^Maternal-Infant Care Research Centre, Mount Sinai Hospital, Toronto, ON, Canada

**Keywords:** anti-VEGF injection, incidence, risk factor, retinopathy of prematurity, treatment

## Abstract

**Purpose:**

To investigate the current incidence, risk factors and treatment for severe retinopathy of prematurity (ROP) in very preterm infants in China.

**Methods:**

This was a descriptive, retrospective cohort study. The study population was very preterm infants admitted to one of the 70 neonatal intensive care units (NICUs) in the Chinese Neonatal Network who underwent ROP screening between January 2019 and December 2020. The primary outcome was severe ROP, defined as type 1 or type 2 ROP. Potential risk factors were identified based on prior evidence and statistical significance. Adjusted odds ratios with 95% confidence intervals were calculated using a multivariable multinomial logistic regression model.

**Results:**

This study included 14,670 very preterm infants, among which 17.3% had low-grade ROP, and 8.5% had severe ROP. Treatment was performed in 1,381 (4.7%) eyes of the infants, among which 1,029 (74.5%), 148 (10.7%), and 124 (9.0%) received intravitreal anti-vascular endothelial growth factor injection only, laser treatment only, and vitreoretinal surgery, respectively. Extremely preterm birth (7.61, 95% CI: 6.12–9.46), small for gestational age (SGA; 2.31, 95% CI: 1.71–3.12), outborn status (1.43, 95% CI: 1.22–1.67), 1-min Apgar score < 7 (1.30, 95% CI: 1.11–1.51), >40 days on oxygen (1.70, 95% CI: 1.37–2.10), >28 days on parenteral nutrition (PN; 1.17, 95% CI: 1–1.35), and any inotropes use (2.12, 95% CI: 1.82–2.46) were associated with increased odds of severe ROP. Antenatal steroid use (0.77, 95% CI: 0.66–0.91), large for gestational age (LGA; 0.62, 95% CI: 0.45–0.85), and surfactant treatment (0.75, 95% CI: 0.64–0.87) were associated with reduced odds of severe ROP.

**Conclusion:**

The incidence of severe ROP in very preterm infants in China was high compared to the developed countries, and intravitreal anti-VEGF injection was the preferred treatment for severe ROP.

## Introduction

Retinopathy of prematurity (ROP) is a severe vaso-proliferative disease of the retina that affects very preterm infants and is a foremost cause of visual impairment or even blindness. In 2010, an estimated 184,700 babies worldwide developed some stage of ROP, with 20,000 of these neonates becoming blind or severely visually impaired (1). ROP is a multifactorial disease influenced by multiple prenatal and postnatal factors, including maternal hypertensive disorders, the mode of birth, gestational age (GA), parenteral nutrition (PN), respiratory management of infants, and neonatal infection control (2). Ophthalmologists may supply different treatments, such as laser photocoagulation and intravitreal anti-vascular endothelial growth factor (anti-VEGF) injection, based on the severity of ROP, the cost, and available resource. From 2008 to 2018, US hospitals participating in the Vermont Oxford Network showed an increase in anti-VEGF treatment and a concomitant reduction in retinal ablation (2).

In 2014, the Chinese Ophthalmological Society released guidelines for ROP screening in China, but national data on ROP remain limited. It was reported that in 1,099 infants with birth weight (BW) < 1,000 g in southern China from 2004–2018, 50.7% of the infants had ROP, and 29.9% had severe ROP (3). Data from the “Outcomes of extremely preterm infants in China 2010–2019” study, which included 3,756 infants with GA < 28 weeks, showed an ROP incidence of 61.8% (2,320) and an overall treatment incidence of 12.6% (4). However, there is a lack of national-level data on the incidence and treatment practices of ROP in very preterm infants, highlighting the urgent need for a national-level cohort study to better perform patient counseling and develop perinatal-neonatal quality improvement strategies. Therefore, this study aims to investigate the incidence rate, risk factors, and evolving treatment of ROP among very preterm infants in a large cohort which included 70 tertiary hospitals from 29 provinces of China.

## Methods

### Study population

This retrospective cohort study included infants who were born before 32 weeks of gestation and admitted to the 70 tertiary-level neonatal intensive care units (NICUs) from the Chinese Neonatal Network (CHNN) in 2019–2020 (5, 6). All participating NICUs were grade A level III NICUs authorized by the Health Administration of China. The sites were selected to provide a large representative cohort of China. The following exclusion criteria were applied: (1) infants with major congenital anomalies, (2) infants who died or were discharged before ROP screening, (3) infants with missing data of birth or ROP screening data, or (4) infants who did not undergo ROP screening.

### Data sources and collection

Trained data abstractors were accountable for data acquisition in each hospital. Data were directly entered into a customized database equipped with built-in error checking, using a standard manual of operations and definitions. Data were transmitted to the CHNN coordinating center electronically in the Children's Hospital of Fudan University, with patient's identity kept confidential. Data quality audit using data re-abstraction was performed annually (7).

Ethics approval was obtained from the Ethics Committee of the Children's Hospital of Fudan University (#CHFU 2018–296). All participating NICUs approved the development, data transfer, hosting, and analysis of the CHNN dataset.

### Ophthalmic examinations and definition of ROP

Preterm infants meeting the screening criteria underwent their first examination between 4 and 6 weeks of life, and follow-up ophthalmic examinations were achieved. The International Classification of ROP guidelines were used to record the location by zone, stage of the disease, and signs of plus disease for each eye (8). The data were analyzed for the most advanced stage of ROP in the eyes of each infant. ROP was categorized as severe ROP (type 1 or type 2 ROP) or low-grade ROP (not meeting type 1 or type 2 criteria) (9, 10). Type 1 ROP was defined as zone I (any stage ROP with plus disease), zone I (stage 3 ROP without plus disease), or zone II (stage 2 or 3 ROP with plus disease). Type 2 ROP was defined as either zone I (stage 1 or 2 ROP without plus disease), or zone II (stage 3 ROP without plus disease). ROP of stage 4 or 5 was also classified as severe ROP, which generally required treatment. Aggressive posterior retinopathy of prematurity (AP-ROP) was a severe form of ROP characterized by rapid progression to advanced stages in posterior ROP. Therefore, AP-ROP was also classified as a severe ROP (10). ROP treatment included any form of therapy (anti-VEGF injection, laser treatment, vitreoretinal surgery) performed by the local ophthalmologist.

### Definition of the maternal and infant characteristics

Maternal hypertension was defined as either (1) new-onset or superimposed on pre-existing hypertension or (2) pre-eclampsia or eclampsia (11). Maternal diabetes was defined as either pre-gestational or gestational diabetes (12). Chorioamnionitis encompassed both histologic and clinical chorioamnionitis. Premature rupture of membranes (PROM) was defined as rupture of membranes over 24 h before birth (13). Antenatal corticosteroid was defined as maternal administration of at least one dose of dexamethasone or betamethasone prior to the delivery. Outborn status was defined as the newborn transferred from the other hospital.

Small for gestational age (SGA) and large for gestational age (LGA) were defined using the Fenton growth charts, which considered GA and sex. SGA was defined as BW below the 10th percentile, while LGA was defined as a birthweight above the 90th percentile (14). Respiratory distress syndrome (RDS) was diagnosed based on (1) a chest x-ray report, (2) clinical confirmation by a neonatologist based on symptoms and signs, or (3) the administration of exogenous surfactant within 2 h of birth. Hemodynamically significant patent ductus arteriosus (hsPDA) was defined as moderate and large PDA (15). Intraventricular hemorrhage (IVH) was defined as grade III or higher, according to Papile's criteria (16). Periventricular leukomalacia (PVL) was diagnosed based on the identification of periventricular cysts on cranial ultrasound or magnetic resonance imaging scans conducted before discharge (17). Necrotizing enterocolitis (NEC) was defined as stage II or above according to the modified Bell's criteria (18). Early or late sepsis was diagnosed based on positive blood culture results indicating sepsis (19). Bronchopulmonary dysplasia (BPD) was defined as oxygen requirement at discharge or 36 weeks post-menstrual age (20). Mortality was defined as neonatal death during the hospitalization of NICU.

### Statistical analysis

The incidence of each type of ROP among very preterm infants was initially summarized based on GA and BW. The treatment rate for each ROP case was calculated on a per-eye basis. Subsequently, the characteristics of preterm infants were compared across three groups (No ROP, low-grade ROP, and severe ROP) using appropriate statistical tests: analysis of variance for normally distributed variables, the Chi-square test for categorical variables, and the Kruskal–Wallis test for highly skewed variables.

To determine the risk factors associated with severe ROP, we conducted a univariable multinomial logistic analysis since infants were classified into three groups by different types of ROP. The selection of risk factors for low-grade/severe ROP was based on both statistical significance (*P* < 0.10) in the univariable logistic regression and previous evidence, including maternal hypertension, antenatal steroid use, gestational age, multiple births, birthweight percentile, Cesarean delivery, outborn status, 1-min Apgar score, days on oxygen support, days on parenteral nutrition, surfactant treatment, PDA, and inotrope use (21). Subsequently, we performed a multivariable multinomial logistic regression analysis to determine the adjusted odds ratios of each risk factor with 95% confidence intervals. Additionally, a sensitivity analysis was conducted to evaluate risk factors associated with any ROP using binary logistic regression analyses.

Data management and statistical analyses were carried out using SAS Version 9.4 (SAS Institute, Cary, North Carolina), with the statistical significance level set at *P* < 0.05 using a two-tailed test.

## Results

### Study population and incidence of ROP in very preterm infants in China

**Figure 1:**
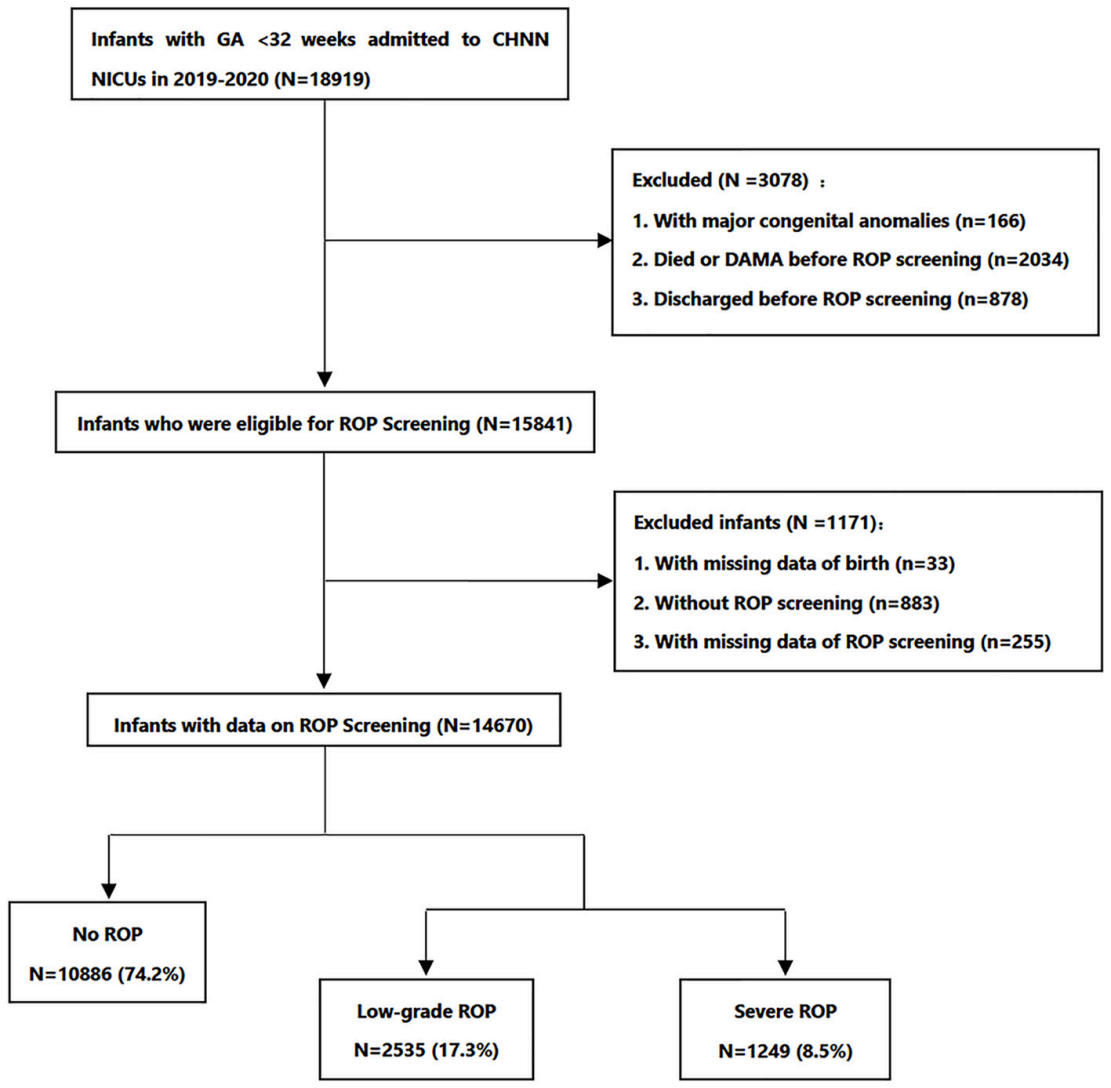
Study flowchart. ROP, retinopathy of prematurity; DAMA, discharged against medical advice.

During the study period, 18,919 infants with GA at birth less than 32 weeks were admitted to 70 NICUs, and 15,841 infants (83.7%) were eligible for ROP screening. Study flowchart was shown in Figure 1. The overall incidence of any grade of ROP was 25.8% (3,784/14,670; Table 1). There was an increase in ROP incidence in infants born at earlier gestational ages, from 78.8% (67/85) among infants born before 25 weeks of gestation to 11.4% (424/3,729) among those born at 31 weeks of gestation (Table 1). The incidence of severe ROP decreased from 50.6% (43/85) among infants born before 25 weeks of gestation to 3.6% (136/3,729) among those born at 31 weeks of gestation (Table 1). Among 4,095 infants with GA < 32 weeks and BW ≥1,500 g, 10.9% were diagnosed with ROP, and 3.7% had severe ROP (Table 1).

**Table 1:** Incidence of retinopathy of prematurity by gestational age and birth weight.

**Characteristics**	**No ROP**	**Low-grade ROP**	**Severe ROP**	**Any ROP**
**GA (weeks)**				
≤ 24 (*n* = 85)	18 (21.2%)	24 (28.2%)	43 (50.6%)	67 (78.8%)
25–26 (*n* = 869)	264 (30.4%)	335 (38.6%)	270 (31.1%)	605 (69.6%)
27–28 (*n* = 3,490)	2,066 (59.2%)	989 (28.3%)	435 (12.5%)	1,424 (40.8%)
29–30 (*n* = 6,497)	5,233 (80.5%)	899 (13.8%)	365 (5.6%)	1,264 (19.5%)
31 (*n* = 3,729)	3,305 (88.6%)	288 (7.7%)	136 (3.6%)	424 (11.4%)
**BW (g)**				
< 750 (*n* = 242)	59 (24.4%)	88 (36.4%)	95 (39.3%)	183 (75.6%)
750–999 (*n* = 1,814)	840 (46.3%)	599 (33.0%)	375 (20.7%)	974 (53.7%)
1,000–1,249 (*n* = 4,012)	2,697 (67.2%)	922 (23.0%)	393 (9.8%)	1,315 (48.8%)
1,250–1,499 (*n* = 4,507)	3,642 (80.8%)	630 (14.0%)	235 (5.2%)	865 (23.8%)
≥1,500 (*n* = 4,095)	3,648 (89.1%)	296 (7.2%)	151 (3.7%)	447 (10.9%)
Total (*n* = 14,670)	10,886 (74.2%)	2,535 (17.3%)	1,249 (8.5%)	3,784 (25.8%)

GA, gestational age; BW, birth weight ROP; retinopathy of prematurity.

### Risk factors of severe ROP among very preterm infants in China

Infants with severe ROP were less likely to have received antenatal corticosteroids compared to those without ROP (Table 2). There was an increased trend in the SGA rate with the severity of ROP, from 4.3% among those without ROP to 6.5% among those with severe ROP. There was an increased trend of outborn and decreased C-section rates with an increased severity of the ROP (Table 2).

**Table 2:** Comparison of maternal and infant's characteristics between the three groups.

**Characteristics**	**No ROP (*n* = 10,886)**	**Low-grade ROP (*n* = 2,535)**	**Severe ROP (*n* = 1,249)**	***P* value**
**Maternal characteristics**				
Maternal age (years), Mean (std)	31.08 (4.95)	31.15 (4.85)	31.25 (4.97)	0.43^a^
Maternal hypertension, *n*/*N* (%)	2,130/10,758 (19.8)	478/2,500 (19.1)	208/1,223 (17.0)	0.06^b^
Maternal diabetes, *n*/*N* (%)	1,981/10,758 (18.4)	459/2,494 (18.4)	229/1,219 (18.8)	0.94^b^
Chorioamnionitis, *n*/*N* (%)	1,488/8,831 (16.8)	352/1,894 (18.6)	155/867 (17.9)	0.17^b^
PROM >24 h, *n*/*N* (%)	2,472/10,249 (24.1)	529/2,339 (22.6)	250/1,126 (22.2)	0.14^b^
Any antenatal steroid use, *n*/*N* (%)	7,935/10,006 (79.3)	1,708/2,235 (76.4)	760/1,073 (70.8)	< 0.01^b^
Complete antenatal steroid course, *n*/*N* (%)^d^	5,052/7,514 (67.2)	1,048/1,591 (65.9)	431/686 (62.8)	0.04^b^
**Infant's characteristics**				
GA (weeks), Median (IQR)	30.14 (29.00,31.00)	28.71 (27.57,30.00)	28.29 (26.86,29.86)	< 0.01^c^
BW (g), Mean (std)	1,377.72 (283.30)	1,170.02 (267.17)	1,114.56 (289.57)	< 0.01^a^
SGA (< P10), *n*/*N* (%)	471/10,875 (4.3)	136/2,534 (5.4)	81/1,249 (6.5)	< 0.01^b^
LGA (>P90), *n*/*N* (%)	688/10,875 (6.3)	128/2,534 (5.1)	66/1,249 (5.3)	< 0.01^b^
Male gender, *n*/*N* (%)	6,281/10,875 (57.8)	1,415/2,534 (55.8)	715/1,249 (57.2)	0.21^b^
Multiple births, *n*/*N* (%)	3,202/10,886 (29.4)	844/2,535 (33.3)	399/1,249 (31.9)	< 0.01^b^
Cesarean delivery, *n*/*N* (%)	6,508/10,859 (59.9)	1,335/2,520 (53.0)	592/1,247 (47.5)	< 0.01^b^
Outborn status, *n*/*N* (%)	3,656/10,886 (33.6)	1,105/2,535 (43.6)	609/1,249 (48.8)	< 0.01^b^
Apgar score at 1 min < 7, *n*/*N* (%)	2,106/10,617 (19.8)	790/2,440 (32.4)	403/1,176 (34.3)	< 0.01^b^
Apgar score at 5 min < 7, *n*/*N* (%)	545/10,306 (5.3)	228/2,336 (9.8)	111/1,142 (9.7)	< 0.01^b^
**Neonatal comorbidities and treatments**				
RDS, *n*/*N* (%)	7,273/10,845 (67.1)	1,925/2,512 (76.6)	859/1,233 (69.7)	< 0.01^b^
Surfactant treatment, *n*/*N* (%)	5,270/10,886 (48.4)	1,407/2,535 (55.5)	619/1,249 (49.6)	< 0.01^b^
hsPDA, *n*/*N* (%)	1,445/10,701 (13.5)	591/2,507 (23.6)	282/1,221 (23.1)	< 0.01^b^
IMV, *n*/*N* (%)	4,165/10,886 (38.3)	1,275/2,535 (50.3)	675/1,249 (54.0)	< 0.01^b^
Days on IMV, Median (IQR)^e^	4.0 (2.0,8.0)	7.0 (3.0,15.0)	10.0 (4.0,23.0)	< 0.01^c^
NIV, *n*/*N* (%)	9,807/10,886 (90.1)	2,373/2,535 (93.6)	1,077/1,249 (86.2)	< 0.01^b^
Oxygen, *n*/*N* (%)	7,010/10,886 (64.4)	1,718/2,535 (67.8)	804/1,249 (64.4)	< 0.01^b^
Days on oxygen, Median (IQR)^f^	34.0 (31.0,37.0)	35.0 (31.0,38.0)	35.0 (32.0,38.0)	0.04^c^
BPD, *n*/*N* (%)	2,990/10,886 (27.5)	1,065/2,535 (42.0)	614/1,249 (49.2)	< 0.01^b^
Late sepsis, *n*/*N* (%)	780/10,886 (7.2)	248/2,535 (9.8)	124/1,249 (9.9)	< 0.01^b^
NEC (≥stage II), *n*/*N* (%)	473/10,886 (4.3)	140/2,535 (5.5)	74/1,249 (5.9)	< 0.01^b^
PN, *n*/*N* (%)	10,210/10,886 (93.8)	2,400/2,535 (94.7)	1,082/1,249 (86.6)	< 0.01^b^
Days on PN, Median (IQR)^g^	22.0 (14.0,32.0)	28.0 (18.0,40.0)	31.0 (20.0,45.0)	< 0.01^c^
Any inotropes use, *n*/*N* (%)	2,245/10,886 (20.6)	727/2,535 (28.7)	475/1,249 (38.0)	< 0.01^b^
RBC transfusion, *n*/*N* (%)	5,964/10,886 (54.8)	1,845/2,535 (72.8)	851/1,249 (68.1)	< 0.01^b^
IVH (III-IV)/PVL, *n*/*N* (%)	829/10,298 (8.1)	318/2,426 (13.1)	192/1,141 (16.8)	< 0.01^b^
Length of NICU stay (days), Median (IQR)	44.0 (34.0,56.0)	59.0 (44.0,75.0)	61.0 (42.0,82.0)	< 0.01^c^
Death, *n*/*N* (%)	190/10,886 (1.7)	31/2,535 (1.2)	25/1,249 (2.0)	0.11^b^

PROM, premature rupture of membranes; GA, gestational age; BW, birth weight; SGA, small for gestational age; LGA, large for gestational age; RDS, respiratory distress syndrome; hsPDA, hemodynamically significant patent ductus arteriosus; NEC, necrotizing enterocolitis; IMV, invasive mechanical ventilation; NIV, non-invasive ventilation; PN, parenteral nutrition; RBC, red blood cell; BPD, bronchopulmonary dysplasia; SD, standard deviation; IQR, interquartile range.

a Analysis of variance (ANOVA) was used to produce the P-value.

b Chi-square test was used to produce the P-value.

c Kruskal–Wallis test was used to produce the P-value.

d Rates were calculated among those with any doses of antenatal corticosteroids.

e Median days on IMV were calculated among those with IMV.

f Median days on oxygen were calculated among those with oxygen.

g Median days on PN were calculated among those with PN.

The median GA at birth was 29.86 (IQR: 28.57, 31.00) weeks, and the mean BW was 1,319.42 g (SD: 298.28, Table 2). There were 10,225 singletons (69.7%) and 8,411 males (57.4%) among the study population (Table 2). Infants with severe ROP had a higher rate of 1-min Apgar score of less than 7 compared to those without ROP. In addition, the rate of hsPDA was higher among those with severe ROP than those without ROP. Infants with severe ROP received longer PN and more inotropes than those without ROP (Table 2).

As shown in Table 3, antenatal steroid use was related to the reduced odds of severe ROP (aOR: 0.77, 95% CI: 0.66, 0.91). Compared with the preterm infants born at 30–31 weeks of gestation, the adjusted OR of severe ROP among those born at 28–29 weeks of gestation was 2.11 (95%CI: 1.75, 2.54), which was lower compared to extremely preterm infants with gestation age < 28 weeks (aOR: 7.61, 95% CI: 6.12, 9.46). The adjusted OR of severe ROP for infants with SGA and LGA was 2.31 (95% CI: 1.71 to 3.12), and 0.62 (95% CI: 0.45, 0.85), compared to those with appropriate for gestational age (AGA), respectively. In addition, there were significantly higher odds of severe ROP among outborn infants (aOR: 1.43, CI 1.22, 1.67) than inborn infants. An Apgar score at 1 min < 7 was associated with higher odds of severe ROP (aOR: 1.30, 95% CI: 1.11, 1.51).

**Table 3:** Risk factors for severe retinopathy of prematurity in preterm infants.

**Characteristics**	**Crude OR (95% CI)**		**Adjusted OR (95% CI)**	
	**Low-grade ROP**	**Severe ROP**	**Low-grade ROP**	**Severe ROP**
Maternal hypertension	0.96 (0.86, 1.07)	0.83 (0.71, 0.97)	1.16 (1.01, 1.33)	0.90 (0.73, 1.11)
Any antenatal steroid use	0.85 (0.76, 0.94)	0.63 (0.55, 0.73)	0.94 (0.83, 1.06)	0.77 (0.66, 0.91)
**Gestational age (weeks)**				
≤ 27	7.96 (7.03, 9.01)	11.51 (9.82, 13.49)	5.89 (5.00, 6.94)	7.61 (6.12, 9.46)
28–29	2.62 (2.36, 2.91)	2.45 (2.11, 2.86)	2.35 (2.08, 2.67)	2.11 (1.75, 2.54)
30–31	ref	ref	ref	ref
Multiple births	1.20 (1.09, 1.31)	1.13 (0.99, 1.28)	1.15 (1.03, 1.28)	1.09 (0.94, 1.26)
**BW for GA**				
AGA (P10–90)	ref	ref	ref	ref
SGA (< P10)	1.24 (1.02, 1.50)	1.52 (1.19, 1.94)	1.53 (1.21, 1.92)	2.31 (1.71, 3.12)
LGA (>P90)	0.80 (0.66, 0.97)	0.85 (0.65, 1.10)	0.59 (0.47, 0.75)	0.62 (0.45, 0.85)
Cesarean delivery	0.75 (0.69, 0.82)	0.60 (0.54, 0.68)	1.02 (0.91, 1.14)	0.91 (0.78, 1.06)
Outborn	1.54 (1.41, 1.67)	1.89 (1.67, 2.13)	1.39 (1.25, 1.56)	1.43 (1.22, 1.67)
Apgar score at 1 min < 7	1.93 (1.76, 2.13)	2.11 (1.85, 2.40)	1.25 (1.12, 1.40)	1.30 (1.11, 1.51)
**Days on oxygen**				
0–20	ref	ref	ref	ref
21–40	1.71 (1.52, 1.93)	1.14 (0.96, 1.35)	1.23 (1.07, 1.42)	1.04 (0.84, 1.29)
>40	3.61 (3.23, 4.02)	3.63 (3.15, 4.18)	1.46 (1.26, 1.70)	1.70 (1.37, 2.10)
**Days on PN**				
0–27	ref	ref	ref	ref
≥28	1.89 (1.73, 2.06)	2.05 (1.82, 2.31)	1.19 (1.07, 1.33)	1.17 (1.00, 1.35)
Surfactant treatment	1.33 (1.22, 1.45)	1.05 (0.93, 1.18)	0.99 (0.89, 1.10)	0.75 (0.64, 0.87)
hsPDA	1.98 (1.77, 2.20)	1.92 (1.67, 2.22)	1.23 (1.08, 1.39)	1.08 (0.91, 1.28)
Any inotropes use	1.55 (1.40, 1.71)	2.36 (2.09, 2.67)	1.20 (1.07, 1.34)	2.12 (1.82, 2.46)

GA, gestational age; BW, birth weight; SGA, small for gestational age; AGA, appropriate for gestational age; LGA, large for gestational age; PN, parenteral nutrition; hsPDA, hemodynamically significant patent ductus arteriosus.

Regarding NICU intervention, infants with oxygen support for 40 days or more had significantly higher odds of severe ROP (aOR, 1.70, 95% CI: 1.37, 2.10), than those who received oxygen support for less than 20 days (Table 3). Similarly, infants who received PN for 28 days or more had significantly higher odds of severe ROP (aOR: 1.17, 95% CI: 1.00, 1.35, Table 3). The use of inotropes was associated with higher odds of severe ROP (aOR: 2.12, 95% CI: 1.82, 2.46, Table 3). However, the adjusted OR of severe ROP associated with surfactant treatment was 0.75 (95% CI: 0.64, 0.87, Table 3). The risk factors for low-graded ROP were similar to those for severe ROP (Table 3).

### ROP treatment rate during hospitalization

Of the 29,293 eyes screened for ROP, 1,381 (4.71%) eyes were treated. 1,095 (48.8%) eyes with severe ROP and 279 (5.8%) eyes with low-grade ROP received treatment. Of the 1,381 treated eyes, 1,029 (74.5%) eyes underwent intravitreal anti-VEGF injection only, 148 (10.7%) eyes received laser treatment only, 124 (9.0%) eyes received vitreoretinal surgery only, 16 (1.2%) eyes received combined treatments at different times, and there were missing data about the treatment of 64 (4.6%) eyes (Table 4). In addition, seven eyes without ROP received ROP-like treatment, likely to prevent potential irreversible vision loss from other severe disease processes, such as familial exudative vitreoretinopathy (FEVER; Table 4) (22, 23).

**Table 4:** Severity and treatment modalities of retinopathy of prematurity in the patients' eyes.

**Severity of ROP**	**Total number of eyes**	**No Treatment**	**Only anti-VEGF injection**	**Only laser treatment**	**Only vitreoretinal surgery**	**Combined treatment**	**Unknown treatment type**	**Total Treatment**
		***n*** **(%)**	***n*** **(%)**	***n*** **(%)**	***n*** **(%)**	***n*** **(%)**	***n*** **(%)**	***n*** **(%)**
No ROP	22,240	22,233 (100.0)	5 (0.0)	1 (0.0)	1 (0.0)	0 (0.0)	0 (0.0)	7 (0.0)
Low-grade ROP	4,808	4,529 (94.2)	224 (4.7)	18 (0.4)	25 (0.5)	0 (0.0)	12 (0.2)	279 (5.8)
Severe ROP	2,245	1,150 (51.2)	800 (35.6)	129 (5.7)	98 (4.4)	16 (0.7)	52 (2.3)	1,095 (48.8)
Total, *n* (%)	29,293	27,912 (95.3)	1,029 (3.5)	148 (0.5)	124 (0.4)	16 (0.1)	64 (0.2)	1,381 (4.7)

Anti-VEGF, anti-vascular endothelial growth factor.

## Discussion

This nationwide cohort study provided important insights into the current incidence, risk factors, and treatment of ROP in very preterm infants in China. The results of our study indicated that the incidence of any ROP and severe ROP in very preterm infants remained high, at 25.8 and 8.5%, respectively, and 11.4% of the infants born at 31 weeks of gestation developed ROP. The key risk factors for severe ROP were extremely preterm birth, outborn status, lower 1-min Apgar score, and longer duration of PN and oxygen support. The intravitreal anti-VEGF injection has been the preferred treatment modality for ROP, compared to laser photocoagulation.

The screening criteria for ROP vary worldwide and are influenced by economic development, population composition, and medical infrastructure. This variation is also a significant factor contributing to the large differences in ROP incidence across different countries. The American guideline recommends screening all infants with a BW < 1,500 g or GA < 30 weeks, while the UK guideline recommends screening all infants with a BW < 1,500 g or a GA < 31 weeks (24, 25). The Postnatal Growth and Retinopathy of Prematurity (G-ROP) retrospective cohort study, conducted in Canada and the United States between January 2006 and December 2011, showed that 12.4% infants with BW ≤ 1,500 g or GA ≤ 30 weeks developed type 1 or type 2 ROP (26). The TR-ROP study in Turkey showed 27% had any ROP and 6.7% had severe ROP among the preterm infants with BW ≤ 1,500 g or GA ≤ 32 weeks (27). In this study, we found the incidence of severe ROP in very preterm infants with GA < 32 weeks was 8.5%, and 11.4% of the infants born at 31 weeks of gestation developed ROP, which was higher compared to developed countries. Therefore, quality improvement projects were needed to reduce the ROP incidence of preterm infants in China. A latest Chinese expert consensus recommends that very preterm infants with GA < 32 weeks or BW less than 2,000 g should receive ROP screening (28). Our study reinforces the current Chinese recommendation of ROP screening criteria not excluding the infants with BW >1500 g.

Smaller GA at birth with lower BW is the dominant risk factor of severe ROP, which was proved in the literature (29). Our study also suggested that 1-min Apgar scores less than 7 were associated with a higher ROP risk among very preterm infants, similar to Rudanko's study (30). Furthermore, consistent with other studies, we also found that antenatal corticosteroids were associated with a lower risk of severe ROP and outborn status was associated with a higher risk of severe ROP among very preterm infants (31). Consequently, perinatal quality improvement programs focusing on reducing premature birth, antenatal corticosteroids, intrauterine transport, standardized neonatal resuscitation and stabilization of the preterm infant may contribute to reduce the risks of ROP.

Beyond the perinatal risk factors, neonatal interventions in the NICU are related to the development of ROP. Our study demonstrated that >40 days of oxygen support and the use of inotropes were associated with a higher risk of severe ROP, corroborating previous research findings (32). Longer exposure to and higher volume of parenteral nutrition increase the risk of infections in the preterm baby. Parenteral nutrition duration of 14 days or more was consistent with a significantly higher risk of having any ROP and receiving ROP treatment in Sweden (33). Our study also showed that infants receiving PN 28 days or more had a higher incidence of ROP. Therefore, appropriate oxygen therapy with monitoring and optimizing enteral nutrition support may reduce the incidence of ROP.

Laser and anti-VEGF injection are the primary treatments for ROP, and vitreoretinal surgery is administered by a specialist if there is any significant peripheral retinal traction. Since the 2011 BEAT-ROP trial reporting that intravitreal bevacizumab monotherapy had a significant advantage over conventional laser ablation for the treatment of a subgroup of type 1 ROP, the proportion of intravitreal injection for the treatment of ROP has increased both in developed and in developing area (34, 35). In 2018, the BEAT-ROP trial reported lower failure rates for anti-VEGF injections compared to laser therapy (36). Intravitreal anti-VEGF therapy (59.1%) has replaced laser photocoagulation as the preferred treatment modality at a Canadian tertiary center since mid-2013 (37). In this study, 74.5% of treated eyes received intravitreal anti-VEGF injection only, while 10.7% received laser therapy only. Thus, the rate of intravitreal anti-VEGF injection in China was much higher compared to developed countries, which might be attributed to its ease of administration at the bedside.

In conclusion, the incidence of ROP and severe ROP in very preterm infants in China were high compared to the developed countries, and intravitreal anti-VEGF injection was the preferred treatment modality. These findings offered valuable insights to further develop quality improvement strategies for ROP among preterm infants in China.

## Strengths and weaknesses

There are several strengths in this study. Firstly, it is the first study using national-level data in China with a considerable sample size, which can reflect current perinatal and neonatal care practices for severe ROP. Secondly, most CHNN participating hospitals conducted ROP screenings by ophthalmologists who employed standard International Classification of ROP terminology for diagnosis. Thirdly, our study maintained high data quality related to ROP and other perinatal-neonatal variables through annual data checks, ensuring accurate incidence rates and reliable point estimates in the multivariable regression analysis.

However, this study has several limitations. Firstly, the data was derived from a select group of large tertiary NICUs in China. These NICUs were from capitals or regional referral centers, which were relatively affluent and might not represent very preterm infants in the rural area. Secondly, this was a retrospective analysis, and our database captured data of the patients in a CHNN member hospital. ROP treatment varied significantly between the NICUs in China, some Maternal and Child Health Hospitals do not provide ROP treatment, the patients with type 1 ROP will be transferred to children's hospitals or general hospitals. If the patient received the treatment in the member hospital after discharge, the occurrence or treatment were included. However, the data was not included if the patient was transferred to non-CHNN member hospitals. Thus, the incidence and treatment rate of ROP might be underestimated. Finally, while clinicians aim for oxygen saturation levels of 90%−95% in neonates, evidence shows infants spend less than 50% of the time within this range. This study lacks data on time spent within the target range, highlighting the need for future research on its association with severe ROP.

## Author's note

Portions of this investigation were presented at PAS 2023 (Washing D.C., USA) on May 1, 2023.

## Data Availability

The original contributions presented in the study are included in the article/Supplementary material, further inquiries can be directed to the corresponding author.
