# Supplementary material for: Incidence, risk factors and evolving treatment of severe retinopathy of prematurity in China: a retrospective multicenter cohort study in 70 neonatal intensive care units
Source: Front Med (Lausanne). 2025 Sep 18;12:1652727. doi: 10.3389/fmed.2025.1652727 (PMC12489369; doi:10.3389/fmed.2025.1652727)
Supplement: Supplementary file 1 [file Table_1.docx]

**Web Supplementary Material**

Incidence, risk factors and evolving treatment of severe retinopathy of prematurity in China: a retrospective multicenter cohort study in 70 neonatal intensive care units

**Group Information of the CHNN**

Chairmen: Shoo K. Lee, MBBS, Mount Sinai Hospital, University of Toronto; Chao Chen, MD, Children’s Hospital of Fudan University. Vice-Chairmen: Lizhong Du, MD, Children's Hospital of Zhejiang University School of Medicine; Wenhao Zhou, Children’s Hospital of Fudan University.

Site principle investigators of the Chinese Neonatal Network: Children's Hospital of Fudan University: Yun Cao, MD; The Third Affiliated Hospital of Zhengzhou University: Falin Xu, MD; Tianjin Central Hospital of Obstetrics and Gynecology: Xiuying Tian, MD; Guangzhou Women and Children’s Medical Center: Huayan Zhang, MD; Children’s Hospital of Shanxi: Yong Ji, MD; Northwest Women's and Children's Hospital: Zhankui Li, MD; Gansu Provincial Maternity and Child Care Hospital: Jingyun Shi, MD; Shengjing Hospital of China Medical University: Xindong Xue, MD; Shenzhen Maternity and Child Health Care Hospital: Chuanzhong Yang, MD; Quanzhou Women and Children’s Hospital: Dongmei Chen, MD; The Affiliated Suzhou Hospital of Nanjing Medical University: Sannan Wang, MD; Guizhou Women and Children’s Hospital/Guiyang Children’s Hospital: Ling Liu, MD; Hunan Children’s Hospital: Xirong Gao, MD; The First Bethune Hospital of Jilin University: Hui Wu, MD; Fujian Maternity and Child Health Hospital，Affiliated Hospital of Fujian Medical University: Changyi Yang, MD; Nanjing Maternity and Child Health Care Hospital: Shuping Han, MD; Qingdao Women and Children’s Hospital: Ruobing Shan, MD; The Affiliated Hospital of Qingdao University: Hong Jiang, MD; Children’s Hospital of Shanghai: Gang Qiu, MD; Women and Children's Hospital of Guangxi Zhuang Autonomous Region: Qiufen Wei, MD; Children’s Hospital of Nanjing Medical University: Rui Cheng, MD; Henan Children’s Hospital: Wenqing Kang, MD; The First Affiliated Hospital of Xinjiang Medical University: Mingxia Li, MD; Foshan Women and Children’s Hospital: Yiheng Dai, MD; The First Affiliated Hospital of Anhui Medical University: Lili Wang, MD; Shanghai First Maternity and Infant Hospital: Jiangqin Liu MD; Yuying Children's Hospital Affiliated to Wenzhou Medical University: Zhenlang Lin, MD; Children’s Hospital of Chongqing Medical University: Yuan Shi, MD; The First Affiliated Hospital of Zhengzhou University: Xiuyong Cheng, MD; The First Affiliated Hospital of USTC, Division of Life Sciences and Medicine, University of Science and Technology of China: Jiahua Pan, MD; Shaanxi Provincial People’s Hospital: Qin Zhang, MD; Children's Hospital of Soochow University: Xing Feng, MD; Wuxi Maternity and Child Healthcare Hospital: Qin Zhou, MD; People's Hospital of Xinjiang Uygur Autonomous Region: Long Li, MD; The Second Xiangya Hospital of Central South University: Pingyang Chen, MD; Qilu Children’s Hospital of Shandong University: Xiaoying Li, MD; Hainan Women and Children’s Hospital: Ling Yang, MD; Xiamen Children’s Hospital: Deyi Zhuang, MD; Xinhua Hospital affiliated to Shanghai Jiao Tong University School of Medicine: Yongjun Zhang, MD; Shanghai Children’s Medical Center，Shanghai Jiao Tong University School of Medicine: Jianhua Sun, MD; Shenzhen Children’s Hospital: Jinxing Feng, MD; Children's Hospital Affiliated to Capital Institute of Pediatrics: Li Li, MD; Women and Children’s Hospital, School of Medicine, Xiamen university: Xinzhu Lin, MD; General Hospital of Ningxia Medical University: Yinping Qiu, MD; First Affiliated Hospital of Kunming Medical University: Kun Liang, MD; Hebei Provincial Children's Hospital: Li Ma, MD; Jiangxi Provincial Children’s Hospital: Liping Chen, MD; Fuzhou Children’s Hospital of Fujian Province: Liyan Zhang, MD; First Affiliated Hospital of Xian Jiao Tong University: Hongxia Song, MD; Dehong people's Hospital of Yunnan Province: Zhaoqing Yin, MD; Beijing Children's Hospital，Capital Medical University: Mingyan Hei, MD; Zhuhai Center for Maternal and Child Health Care: Huiwen Huang, MD; Guangdong Women and Children's Hospital: Jie Yang, MD; Dalian Municipal Women and Children’s Medical Center: Dong Li, MD; Peking Union Medical College Hospital: Guofang Ding, MD; Obstetrics & Gynecology Hospital of Fudan University: Jimei Wang, MD; Shenzhen Hospital of Hongkong University: Qianshen Zhang, MD; Children's Hospital of Zhejiang University School of Medicine: Xiaolu Ma, MD
